# Adeno-Associated Virus 2 and Human Adenovirus F41 in Wastewater during Outbreak of Severe Acute Hepatitis in Children, Ireland

**DOI:** 10.3201/eid2904.221878

**Published:** 2023-04

**Authors:** Niamh A. Martin, Gabriel Gonzalez, Liam J. Reynolds, Charlene Bennett, Christine Campbell, Tristan M. Nolan, Alannah Byrne, Sanne Fennema, Niamh Holohan, Sailusha Ratnam Kuntamukkula, Natasha Sarwar, Laura Sala-Comorera, Jonathan Dean, Jose Maria Urtasun-Elizari, Daniel Hare, Emer Liddy, Eadaoin Joyce, John J. O’Sullivan, John M. Cuddihy, Angeline M. McIntyre, Eve P. Robinson, Darren Dahly, Nicola F. Fletcher, Suzanne Cotter, Emer Fitzpatrick, Michael J. Carr, Cillian F. De Gascun, Wim G. Meijer

**Affiliations:** University College Dublin, Dublin, Ireland (N.A. Martin, G. Gonzalez, L.J. Reynolds, C. Bennett, C. Campbell, T.M. Nolan, A. Byrne, S. Fennema, N. Holohan, S.R. Kuntamukkula, N. Sarwar, L. Sala-Comorera, J. Dean, J.M. Urtasun-Elizari, D. Hare, J.J. O’Sullivan, N.F. Fletcher, M.J. Carr, C.F. De Gascun, W.G. Meijer);; Hokkaido University, Sapporo, Japan (G. Gonzalez, M.J. Carr);; Health Protection Surveillance Centre, Dublin, Ireland (E. Liddy, J.M. Cuddihy, A.M McIntyre, E.P. Robinson, S. Cotter);; Irish Water, Dublin, Ireland (E. Joyce);; University College Cork, Cork, Ireland (D. Dahly);; Children’s Health Ireland at Crumlin, Crumlin, Ireland (E. Fitzpatrick)

**Keywords:** adenovirus, hepatitis, viruses, HAdV-F41, AAV2, pediatric hepatitis, wastewater-based epidemiology, Ireland, adeno-associated virus 2, human adenovirus F41, enteric infections

## Abstract

During April–July 2022, outbreaks of severe acute hepatitis of unknown etiology (SAHUE) were reported in 35 countries. Five percent of cases required liver transplantation, and 22 patients died. Viral metagenomic studies of clinical samples from SAHUE cases showed a correlation with human adenovirus F type 41 (HAdV-F41) and adeno-associated virus type 2 (AAV2). To explore the association between those DNA viruses and SAHUE in children in Ireland, we quantified HAdV-F41 and AAV2 in samples collected from a wastewater treatment plant serving 40% of Ireland’s population. We noted a high correlation between HAdV-F41 and AAV2 circulation in the community and SAHUE clinical cases. Next-generation sequencing of the adenovirus hexon in wastewater demonstrated HAdV-F41 was the predominant HAdV type circulating. Our environmental analysis showed increased HAdV-F41 and AAV2 prevalence in the community during the SAHUE outbreak. Our findings highlight how wastewater sampling could aid in surveillance for respiratory adenovirus species.

During April 5–July 8, 2022, the World Health Organization (WHO) received reports of 1,010 probable cases of severe acute hepatitis of unknown etiology (SAHUE) in young children from 35 countries (*1*). Many cases resulted in severe clinical outcomes; ≈5% of patients required liver transplants, and 22 died. Most (48%) cases were reported from the WHO European Region, and 484 cases were reported in previously healthy children from 21 countries (*1*). Ireland reported 28 probable cases of SAHUE in non-A–E hepatitis, including 2 patients who received liver transplants and 1 who died but did not receive a transplant (*2*). 

The causes of SAHUE remain unclear. A possible connection with SARS-CoV-2 has been suggested (*3*), but SARS-CoV-2 RNA was detected in only 16% of SAHUE cases reported in the WHO European Region (*1*). Recent studies suggest a possible association between SAHUE and human adenovirus (HAdV) species F type 41 (HAdV-F41) and adeno-associated virus 2 (AAV2) infections (*4*,*5*; A. Ho et al., unpub. data, https://doi.org/10.1101/2022.07.19.22277425).

HAdVs are double-stranded DNA viruses that cause a wide range of self-limiting illnesses, including upper respiratory infections, conjunctivitis, and gastroenteritis. HAdV infections are particularly common in children because of a lack of humoral immunity, and rare manifestations of HAdV illnesses involving hepatitis and liver failure have been reported, mainly in immunocompromised patients (*6*). To date, HAdV is the most frequently detected pathogen (52%) among SAHUE cases where data are available in Europe (*1*). Furthermore, clinical signs and symptoms of SAHUE can occur several weeks after an acute gastrointestinal episode (A. Ho et al., unpub. data). HAdV-F41 and HAdV-F40 are leading causes of diarrhea worldwide, behind only rotavirus (*7*,*8*). Phylogenetic analysis of specimens taken from SAHUE patients in the United States identified 3 distinct enteric HAdV-F41 variants (*4*). In Europe, HAdV-F41 was also identified in SAHUE cases where sequencing data were available (*1*).

In addition, 2 independent studies in the United Kingdom, identified AAV2 from probable cases (A. Ho et al., unpub. data; S. Morfopoulou et al., unpub. data, https://doi.org/10.1101/2022.07.28.22277963). High detection rates of AAV2 DNA have been reported in whole blood, plasma, and explanted liver tissue in SAHUE cases and, of note, AAV2 was not detected in controls of HAdV-negative and severe hepatitis cases not meeting the SAHUE case definition (A. Ho et al., unpub. data; S. Morfopoulou et al., unpub. data). AAV2 possesses a single-stranded DNA genome of 4.7 kb and requires co-infection with a helper virus, most frequently HAdV but also human herpesvirus (e.g., HHV-6/7) or human papillomavirus, to enable productive replication. AAVs are not known to be associated with human pathology and are intensively studied as gene-delivery vectors although transient hepatitis has been reported in clinical trials (*9*). Subclinical seroconversion occurs early in life, and >90% of adults have antibodies to AAV, showing that the virus is common and widely distributed (*10*,*11*).

AAV2 could represent the primary pathogen in SAHUE cases or serve as a valuable biomarker for HAdV infection (A. Ho et al., unpub. data). Alternatively, the pathology of SAHUE could be the result of co-infection with HAdV-F41 and AAV2, where AAV2 might replicate within HAdV-F41–infected cells outside the liver before causing an abortive infection of hepatocytes (*12*). A recent study identified a possible immunogenetic association on the basis of high frequency of the class II HLA-DRB1*04:01 allele associated with the UK SAHUE cases (A. Ho et al., unpub. data). This allele is more frequently detected in populations in northwestern Europe (*13*), suggesting that children in Ireland could be particularly vulnerable to SAHUE. Nonetheless, because the precise cause of this outbreak has not been identified, monitoring for changes in the prevalence of potential pathogens in the community is increasingly pertinent. Studies have shown that HAdV and AAV2 are detectable in stool samples from persons infected with those viruses (*14*,*15*), suggesting that both viruses could be quantified by using wastewater surveillance.

Wastewater-based epidemiology is a valuable tool for monitoring the prevalence of viral pathogens circulating at the population level (*16*,*17*). During the COVID-19 pandemic, wastewater-based surveillance proved to be an efficient tool for monitoring SARS-CoV-2 infections and identifying circulating variants of concern in Ireland and elsewhere (*18*–*20*). A recent study reported SAHUE clinical cases increased concomitant with an increase the levels of HAdV-F40 and HAdV-F41 detected in wastewater in Northern Ireland (*21*). We hypothesized that HAdV-F and AAV2 could be directly or indirectly associated with the SAHUE outbreak; thus, levels of these viruses in wastewater should positively correlate with the onset of the SAHUE outbreak. We used molecular approaches and next-generation sequencing (NGS) to quantify HAdV-F, AAV2, and SARS-CoV-2 in wastewater and identify HAdV types circulating before and during the peak of SAHUE cases in Ireland.

## Materials and Methods

### Wastewater Sample Collection and Concentration

The Ringsend Wastewater Treatment Plant (WWTP) in Dublin, Ireland, has a capacity of 1.98 million population equivalents and serves the greater Dublin area, which is home to ≈40% of the population of Ireland. Eighty-five 24-hour composite wastewater samples were collected during June 2020–August 2022 and concentrated as previously described (*18*).

### Nucleic Acid Extraction

We extracted DNA and RNA from 250 μL of wastewater concentrate by using QIAcube Connect (QIAGEN, https://www.qiagen.com). We used the DNeasy PowerSoil Pro Kit (QIAGEN) to extract DNA, and the RNeasy PowerMicrobiome Kit (QIAGEN) to extract RNA, according to the manufacturer’s guidelines.

### PCR Quantification of HAdV-F and SARS-CoV-2 in Wastewater

We used TaqMan probe assays and conducted quantitative PCR (qPCR) on the Lightcycler 96 platform (Roche Diagnostics, https://www.roche.com) to quantify DNA from HAdV-F and the human biomarker crAss_2 DNA in wastewater. We selected the HAdV-F species assay to specifically detect HAdV-F40 and HAdV-F41 serotypes by targeting the long-fiber coding gene (*22*). We quantified the crAss_2 bacteriophage as a DNA extraction control in all samples (*23*). We conducted amplification by using FastStart Essential DNA Probes Master (Roche Diagnostics) in a total volume of 20 μL according to manufacturer’s recommendations. We quantified SARS-CoV-2 RNA on the same platform by using LightCycler Multiplex RNA Virus Master (Roche Diagnostics) for reverse transcription qPCR (qRT-PCR), as previously published (*18*). We analyzed all samples in triplicate along with positive and negative controls. We quantified gene targets by a standard curve, which we generated by using a 10-fold serial dilution of gBlock Gene Fragment (Integrated DNA Technologies, https://www.idtdna.com) for the HAdV-F assay (*22*) and targeting HAdV-F41 (GenBank accession no. AB728839), and the control plasmid for the SARS-CoV-2 nucleocapsid 1 (N1) assay from the US Centers for Disease Control and Prevention (*24*,*25*). We provide information on thermocycler conditions and final oligonucleotide primer and probe concentrations (Appendix Table 1).

### Digital PCR Quantification of AAV2 in Wastewater 

We quantified AAV2 DNA in wastewater by using digital PCR (dPCR) on the QIAcuity Four Platform System (QIAGEN) by using 2 separate assays targeting the capsid protein viral protein (VP) 1 and nonstructural protein (NSP) genes (Appendix Table 1). We analyzed samples in duplicate on 26K 24-well Nanoplates (QIAGEN) by using the QIAcuity OneStep Advanced Probe Kit (QIAGEN). We conducted amplification in 40 μL reactions containing 5 μL DNA template and 10 µL master mix (Appendix Table 1). We included positive and negative controls on each nanoplate to determine the fluorescence intensity threshold (Appendix Figure 1). We used AAV2-positive samples as positive controls and nuclease-free water as a negative control. We analyzed results by using QIAcuity Suite software version 2.1 (QIAGEN).

### Nanopore Sequencing of the HAdV Hexon from Wastewater

We further analyzed 12 wastewater samples and 1 clinical stool sample from an HAdV-F41 gastrointestinal case by targeting an ≈800-bp subgenomic fragment from the HAdV hexon-coding gene, which contains type-specific epitopes within variable loop 1, which enables molecular typing. We amplified the fragments by using an established pan-adenovirus endpoint PCR (*26*) and deep sequenced amplicons by using the GridION nanopore device (Oxford Nanopore Technologies, https://nanoporetech.com) on genomic DNA by using the Ligation Sequencing Kit (Oxford Nanopore Technologies) and Native Barcodes (Oxford Nanopore Technologies) (Appendix). We published the raw sequence data in the National Center for Biotechnology Information Sequence Reads Archive (https://www.ncbi.nlm.nih.gov/sra) under BioProject no. PRJNA885073.

### Sequence Analysis

We assessed HAdV types in each sample by aligning the reads against a database of hexon proteins of known types by using Diamond version 2.0.14.152 (*27*) (Appendix Table 2). We used genome sequences from the most frequently aligned types (>5% of reads per sample) as references to assemble consensus sequences for the target fragment by using minimap2 version 2.23 (*28*) and polished contig sequences by using medaka version 1.5.0 (Oxford Nanopore Technologies). We used MAFFT (*29*) for multiple sequence alignment of consensus sequences and reference sequences. We inferred a phylogenetic tree by using RAxML (*30*) and estimated topology support by using a bootstrap approach with 100 repetitions. In addition, we assessed detectable SNPs in each sample by using loFreq version 2 (*31*). We deposited the assembled sequences in GenBank (accession nos. OP554817–910).

### Clinical Data

We obtained publicly available SAHUE probable case data from the Health Protection Surveillance Centre (HPSC) via an HPSC report on acute hepatitis of unknown etiology (*2*). The HPSC defined probable cases as cases in persons <16 years of age with signs and symptoms of SAHUE, including acute hepatitis of unknown etiology (non–hepatitis A–E) with no other likely cause identified, and with serum transaminases (aspartate transaminase or alanine aminotransferase) >500 U/L, who were identified after October 1, 2021 and notified under Infectious Disease (Amendment) (No. 3) Regulations 2003 (regulation 14) (*32*). The National Virus Reference Laboratory provided clinical data from all HAdV-F–positive cases tested as part of routine gastroenteritis screening during July 2020–July 2022. Routine screening is conducted on all samples received from persons with viral gastrointestinal symptoms. Routine screening is standard in Ireland and is applied to all age groups but predominantly represents pediatric gastrointestinal cases.

### Data Analysis

We determined the daily HAdV-F, AAV2, SARS-CoV-2, and crAssphage load flowing through the Ringsend WWTP by using the concentration of each viral marker in genome copies per 100 milliliter (GC/100 mL) and the daily WWTP flow rate of cubic meters per second (m^3^/s); thus, flow rate (m^3^/day) × GC/100 mL × 10^4^ = GC/day. Irish Water (https://www.water.ie) provided WWTP flow rate data. We used log_10_ transformed qPCR and dPCR data and Spearman correlation (*r_s_*) analysis on Prism 9.4.1 (GraphPad Software Inc., https://www.graphpad.com) to examine the correlation between variables over time and considered p<0.001 statistically significant.

## Results

### SAHUE Clinical and Demographic Data

During October 2021–August 2022, Ireland reported 28 probable SAHUE cases in children <12 years of age (Table 1). Among case-patients, 1 death and 2 liver transplants were reported for 3 patients. Overall, 52% (14/27) of tested patients had evidence of HAdV infection and 63.6% (14/22) had evidence of AAV2 infection. Among 25 case-patients tested, 15 (60%) had reports of current or past SARS-CoV-2 infection (*2*).

**Table 1:** Clinicodemographic characteristics of probable SAHUE cases in children during time in which AAV2 and HAdV-F41 were detected in wastewater, Ireland*

Characteristics	No. (%) cases
Age, y	
<1	2
1–4	15
5–11	10
12–16	1
Sex	
M	13
F	15
Ethnicity	
White Irish	27
Other	1
Liver transplant	
Y	2
N	26
International travel	
Y	6
N	18
Unknown	4
SARS-CoV-2 vaccination status	
Vaccinated	2
Unvaccinated	19
Unknown	7
Hospitalization status	
Non-ICU	21
ICU	6
Not hospitalized	1
Positive cases	
HAdV	27 (52)
AAV2	22 (63.6)
SARS-CoV-2†	25 (60)

*Information obtained from Health Protection Surveillance Centre public website on September 23, 2022 (https://www.hpsc.ie/a-z/hepatitis/acutehepatitisofunknownaetiology/title-22079-en.html). AAV2, adeno-associated virus type 2; HAdV, human adenovirus; HAdV-F41, human adenovirus type F41; ICU, intensive care unit; SAHUE, severe acute hepatitis of unknown etiology. †Positive for SARS-CoV-2 antibodies.

### HAdV-F and AAV2 Wastewater Surveillance Over Time

**Figure 1:**
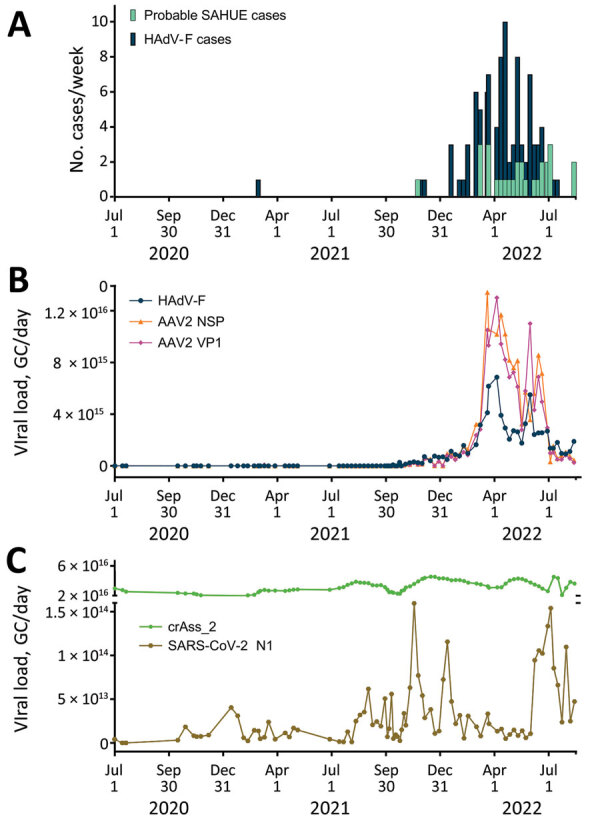
Relationship between AAV2 and HAdV-F in wastewater samples and SAHUE clinical cases in children, Ireland, July 1, 2020–July 30, 2022. Eighty-five 24-h composite wastewater samples collected during July 1, 2020–July 30, 2022, were retroactively analyzed for SARS-CoV-2, AAV2, HAdV-F, and the human biomarker crAss-2. A) Number of probable SAHUE cases and HAdV-F–positive clinical samples reported per week during the study period. B) Daily viral load of HAdV-F, AAV2 VP1, and AAV2 NSP detected in influent of the Ringsend WWTP, Dublin, Ireland. C) Daily viral load of SARS-CoV-2 RNA N1 and DNA extraction crAss_2 control in influent of the Ringsend WWTP. AAV2, adeno-associated virus type 2; GC, genome copies; HAdV-F, human adenovirus types F; N1, nucleocapsid protein 1; NSP, nonstructural protein; SAHUE, severe acute hepatitis of unknown etiology; VP, viral protein; WWTP, wastewater treatment plant.

**Figure 2:**
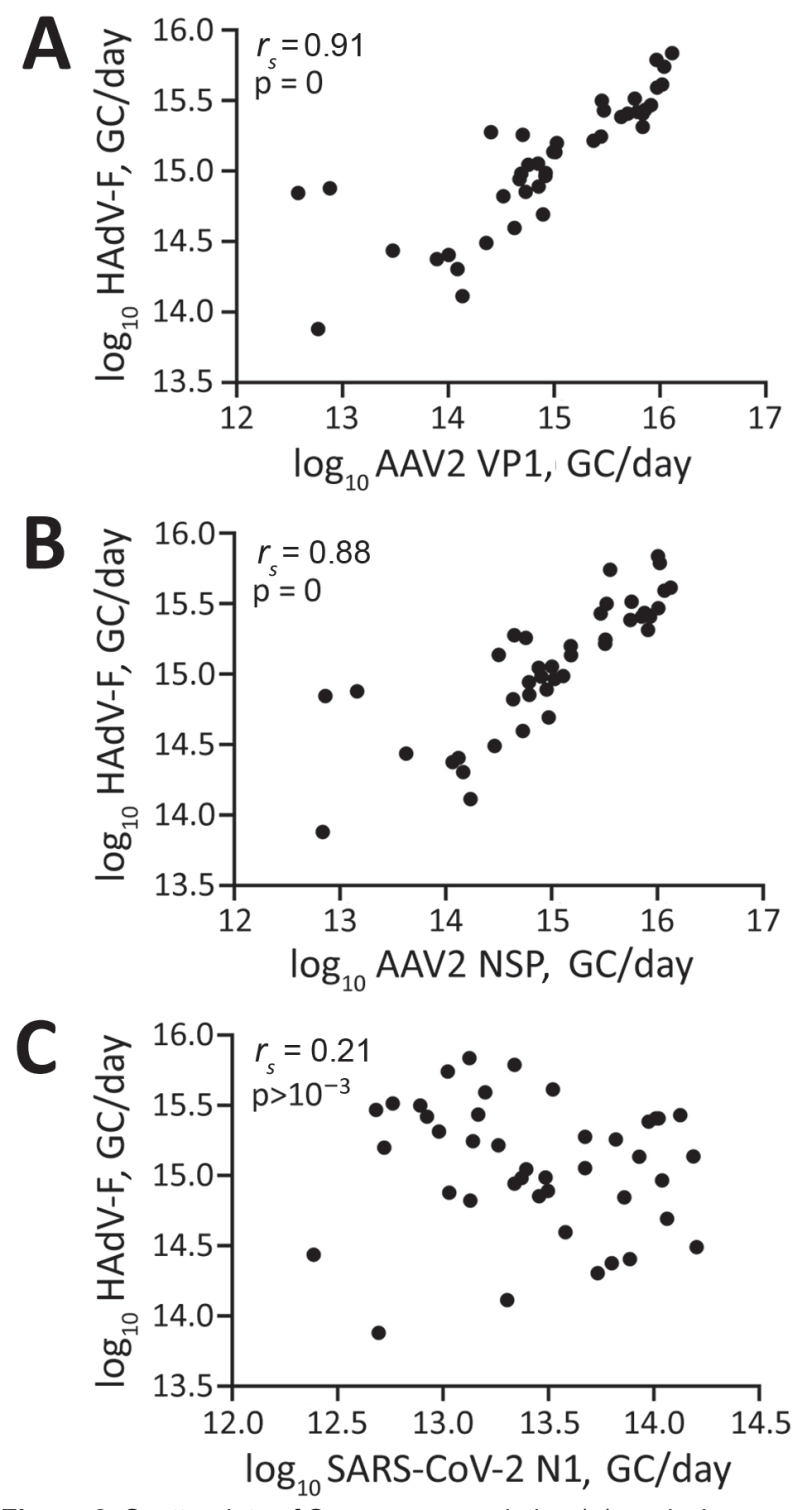
Scatterplots of Spearman correlation (*r_s_*) analysis between HAdV-F, AAV2, and SARS-CoV-2 detected in wastewater samples during outbreak of SAHUE in children, Ireland. Plots depict Spearman correlations between the log_10_ transformed daily HAdV viral load and AAV2 VP1 (A), AAV2 NSP (B), and SARS-CoV-2 N1 (C) in wastewater. AAV2, adeno-associated virus type 2; GC, genome copies; HAdV-F, human adenovirus type F; N1, nucleocapsid protein 1; NSP, nonstructural protein; SAHUE, severe acute hepatitis of unknown etiology.

We retrospectively screened 85 wastewater samples to determine HAdV-F and AAV2 viral load in wastewater in the greater Dublin area during June 2020–August 2022. We detected HAdV-F in wastewater collected on October 13, 2021, at 7.6 × 10^13^ GC/day. HAdV-F levels remained low, but on April 6, 2022, levels increased by 2 orders of magnitude and peaked at 6.86 × 10^15^ GC/day. Of note, AAV2 followed a similar trend, also peaking on April 6, 2022, for VP1 at 1.3 × 10^16^ GC/day; NSP peaked on March 21, 2022, at 1.34 × 10^16^ GC/day (Figure 1, panel B). HAdV-F levels in wastewater significantly correlated with both AAV2 VP1 (*r_s_* = 0.91; p = 0) and NSP (*r_s_* = 0.88; p = 0) genes in wastewater (Figure 2).

The daily concentration of SARS-CoV-2 RNA was quantified in wastewater throughout the sampling period (*18*). SARS-CoV-2 levels in wastewater fluctuated over time, peaking in November 2021 and January 2022, during the 4th and 5th waves of the SARS-CoV-2 pandemic, then decreased in February 2022. A 3rd peak was observed in July 2022. We observed no significant correlation between SARS-CoV-2 and HAdV-F (*r_s_* = –0.21, p>0.001) (Figure 2, panel C) or AAV2 (VP1 *r_s_* = 0.28, p>0.001; NSP *r_s_* = 0.28, p>0.001) over time (Table 2).

**Table 2:** Spearman correlations between clinical SAHUE cases in children and daily viral loads of AAV2, HAdV, and SARS-CoV-2 in wastewater, Ireland*

Correlations between detected viruses	Spearman *r_s_* (95% CI)
No. SAHUE probable cases/week	
HAdV-F GC/day	**0.62** (0.382–0.781)
AAV2 VP1 GC/day	**0.57** (0.323–0.740)
AAV2 NSP GC/day	**0.55** (0.297–0.727)
SARS-CoV-2 N1 GC/day	0.006 (−0.291 to 0.303)
No. HAdV-F clinical cases/week	
HAdV-F GC/day	**0.85** (0.719–0.920)
AAV2 VP1 GC/day	**0.82** (0.723–0.884)
AAV2 NSP GC/day	**0.81** (0.713–0.880)
SARS-CoV-2 N1 GC/day	0.206 (−0.022 to 0.414)
HAdV-F GC/day	
AAV2 VP1 GC/day	**0.91** (0.833–0.951)
AAV2 NSP GC/day	**0.88** (0.787–0.937)
SARS-CoV-2 N1 GC/day	
HAdV-F GC/day	−0.21 (−0.494 to 0.105)
AAV2 VP1 GC/day	0.28 (0.055–0.483)
AAV2 NSP GC/day	0.29 (0.063–0.492)

*Bold text indicates statistical significance (p<0.001). AAV2, adeno-associated virus type 2; GC, genome copies; HAdV-F, human adenovirus species F; N1, nucleocapsid protein 1; NSP, nonstructural protein; SAHUE, severe acute hepatitis of unknown etiology; VP, viral protein.

### HAdV Types in Wastewater

**Figure 3:**
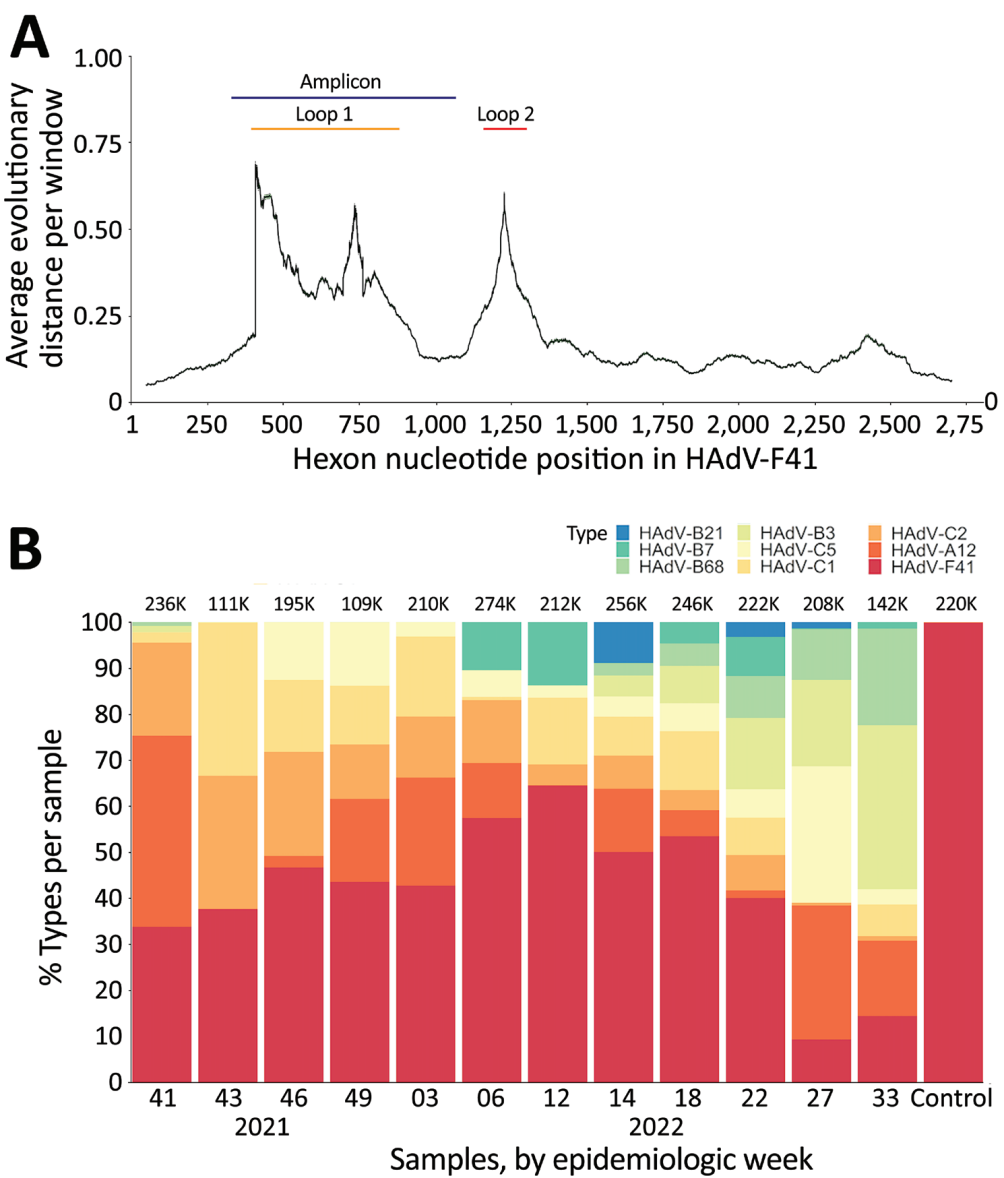
Hexon fragment sequencing for HAdV typing in wastewater, Ireland. A) Diagram of the average nucleotide evolutionary distance among all known HAdV types across the hexon-coding gene. The positions of the variable loops 1 and 2 have been annotated relative to positions in HAdV-F41. The amplicon target used for typing is annotated in blue. B) Type classification of reads in each sample, shown as the percentage of the total of aligned reads per sample, shown at the top of each bar. HAdV, human adenovirus.

The hexon genomic fragment we targeted for deep sequencing encompasses the region between nt positions 17921 and 18661 relative to the HAdV-F41 prototype (GenBank accession no. ON442316), including variable loop 1 of the hexon protein (*26*) (Figure 3, panel A). That fragment enables HAdV type classification based on similarity to other characterized types. The number of aligned reads varied among samples from 111,000–274,000 reads (Figure 3, panel B); however, we detected HAdV-F41 in all samples, supporting the real-time qPCR results we described. Few (<1%) reads aligned with HAdV-F40. Samples collected from week 41 of 2021 (October 13) to week 22 of 2022 (June 1) showed >30% of sequenced reads belonged to HAdV-F41, peaking on week 12 of 2022 (March 23). In contrast, by week 27 (July 5) the proportion of HAdV-F41 fell to 10%. Of note, besides HAdV-F41, the NGS protocol detected other HAdV types circulating in the community, such as HAdV-B3, -B7, -C1, -C2, and C5, which are usually related to respiratory infections (*6*,*33*), and even detected changes in the progression of the proportion among these types.

**Figure 4:**
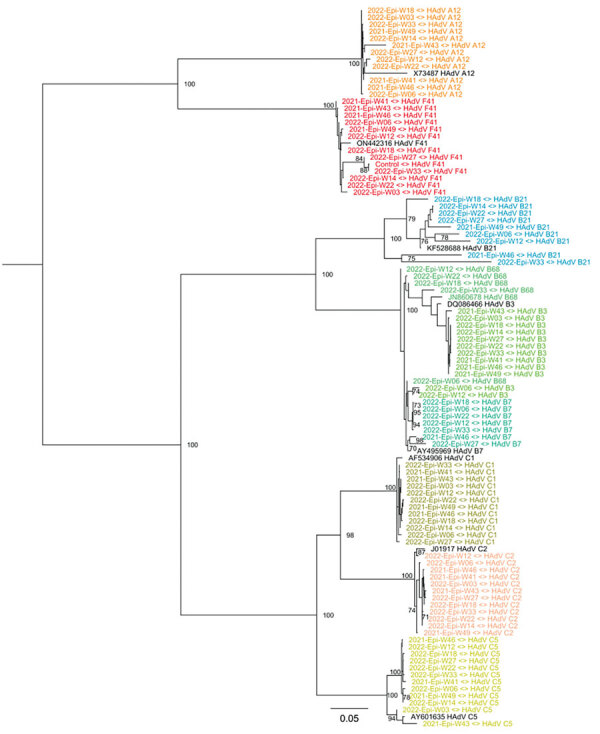
Maximum-likelihood phylogenetic tree of HAdV genetic diversity in wastewater samples collected during SAHUE outbreak in children, Ireland. The tree was inferred by using RAxML (https://github.com/stamatak/standard-RAxML). Branch support was estimated by using the bootstrap method with 100 repetitions and is shown next to the branches that have >70% support. Black text indicates reference sequences, identified by GenBank accession number. Colors indicate HAdV species and types. Scale bar indicates nucleotide substitutions per site. Epi-W, epidemiologic week; HAdV, human adenovirus; SAHUE, severe acute hepatitis of unknown etiology.

We assembled the reads into consensus sequences to assess their genetic relationships. We compared consensus sequences against HAdV type reference sequences in a maximum-likelihood phylogenetic tree that showed well-supported separation among types and consensus sequence clustering that corresponded to different sampling dates (Figure 4). We noted some divergence among consensus sequences in the HAdV-F41 clade, which prompted us to assess single-nucleotide variants (SNVs) in different samples (Appendix Figure 2). Despite finding 46–59 SNVs in samples with a frequency >10%, we observed that 44 SNVs remained constant for all 12 samples, suggesting a rather homogenous population of HAdV-F41 variants circulating in Ireland during the SAHUE outbreak. Nevertheless, the frequency of variants in samples were not constant and we observed some genetic drift over time. For instance, A603G and T604C, relative to the sequenced fragment, increased from a frequency of <25% in week 41 (2021) to >70% by week 33 (2022).

### Correlation of SAHUE and Viral Nucleic Acids in Wastewater Over Time

The clinical reports of SAHUE showed a significant correlation with the HAdV-F viral load detected in wastewater samples collected during October 13, 2021–August 14, 2022 (*r_s_* = 0.62; p<0.001) (Table 2). Similarly, we observed a significant correlation between SAHUE cases and detection of both AAV2 gene targets in wastewater, VP1 (*r_s_* = 0.57; p<0.001) and NSP (*r_s_* = 0.55; p<0.001). In contrast, SAHUE cases showed no correlation to SARS-CoV-2 detection in wastewater samples during the same period (*r_s_* = 0.006; p = 0.97) (Table 2).

### Correlation of HAdV-F Gastrointestinal Cases and HAdV-F DNA in Wastewater Over Time

We evaluated the relationship between the number of reported HAdV-F gastrointestinal cases and HAdV-F DNA levels in wastewater (Table 2; Figure 1). We observed a significant correlation, inferring that HAdV-F DNA levels in wastewater reflect the prevalence of HAdV-F in the community (*r_s_* = 0.85; p<0.001) (Table 2). We also observed a significant correlation between clinical HAdV-F cases and AAV2 VP1 (*r_s_* = 0.82; p<0.001) and AAV2 NSP (*r_s_* = 0.81; p<0.001) (Table 2). In contrast, we detected no significant correlation between HAdV-F and AAV2 and the levels of SARS-CoV-2 N1 in wastewater (*r_s_* = 0.206; p>0.1).

## Discussion

We examined associations between the number of pediatric SAHUE cases reported and the levels of HAdV-F and AAV2 in wastewater influent taken from the Ringsend WWTP in Dublin, Ireland. By September 8, 2022, Ireland had 28 probable SAHUE cases reported (*2*). Probable cases were geographically spread broadly throughout Ireland, but most cases occurred in Dublin, an area served by the Ringsend WWTP, which captures ≈40% of the population of Ireland.

Our results showed a positive temporal correlation between the number of SAHUE cases and the daily viral load of both HAdV-F and AAV2 in wastewater during the outbreak period, consistent with the hypothesis that these 2 viruses could be directly or indirectly involved with the etiopathogenesis of SAHUE. Of 27 probable cases tested for HAdV in Ireland, 14 (52%) tested positive (*2*), which is comparable to other SAHUE cases reported in Europe during that time (*1*). Furthermore, 64% (14/22) of cases analyzed for AAV2 also tested positive.

Our results showed that HAdV-F levels in wastewater corresponded with the trends in the number of HAdV-positive cases reported weekly throughout the study period. A similar robust correlation was seen between the levels of AAV2 in wastewater and clinical HAdV cases reported. Correlation between AAV2 and HAdV-F is expected because of the adenovirus-dependent nature of AAV2. 

In addition to AAV2 and HAdV-F, our results showed other respiratory adenoviruses were circulating in the community. Sequencing results showed a high prevalence of HAdV-F41 and low prevalence of HAdV-F40 in wastewater, suggesting that HAdV-F41 was the dominant F species circulating in the community during the study period. In addition, a large number (52%) of hexon loop 1 reads derived from HAdV species A–E enabled identification of other HAdV species associated with respiratory disease. This finding highlights that wastewater sampling could be used for surveillance of respiratory adenoviral species, just as for surveillance for other respiratory viruses, such as SARS-CoV-2 and influenza (*18*,*34*).

Our results showed no statistically significant relationship between SARS-CoV-2 levels and either HAdV-F and AAV2 in wastewater, suggesting that the epidemiology of the viruses is different. Of note, SARS-CoV-2 was detectable throughout the SAHUE outbreak period. Although we did not observe an association between SARS-CoV-2 levels in wastewater and the number of SAHUE cases, the relationship of SARS-CoV-2 infections with the SAHUE cases remains to be explored. In Ireland, 60% of SAHUE cases also tested positive for SARS-CoV-2 antibodies, indicating a current or past SARS-CoV-2 infection.

Throughout the COVID-19 pandemic, nonpharmaceutical interventions (NPIs), such as mandatory face coverings and social distancing, played a crucial role in reducing SARS-CoV-2 transmission rates. Studies have shown that COVID-19 NPIs were associated with reduced transmission of other viruses, such as HAdV and influenza (*35*–*37*). Our results support those findings; HAdV-F was not detectable in wastewater during July 2020–October 2021, when mandatory NPIs were in place in Ireland. Wastewater samples from before the SARS-CoV-2 pandemic are not available; therefore, the level of HAdV in wastewater before March 2020 remains unknown.

Similarly, AAV2 persisted at lower levels throughout the pandemic before increasing toward the end of 2021. The intensity of the SAHUE outbreak might be a result of the immunologically naive status of young children who had limited exposure to HAdV species and AAV2 during the preceding 2 years.

After February 28, 2022, public health measures such as social distancing and pods were abolished in schools and early learning settings in Ireland; beginning on April 1, 2022, all COVID-19 restrictions ended in the wider community (*38*). Those dates coincide with the sharp increase in the number of weekly HAdV-positive cases and HAdV-F and AAV2 DNA levels in wastewater, indicative of an increase in the transmission of both viruses and a higher number of infections in the community. Furthermore, NGS data showed that the proportion of sequenced reads belonging to HAdV-F41 peaked around that time, suggesting that HAdV-F41 was one of the dominant HAdV serotypes circulating during the study period.

In conclusion, the observed increases in prevalence of HAdV-F41 and AAV2 DNA in wastewater correlated with clinical SAHUE cases in Ireland, supporting the hypothesis that these viruses could be involved in SAHUE outbreaks. Although such correlations are associative and are not direct evidence of causation, our results showing the identity and changes in prevalence of HAdV-F41 and AAV2, provide compelling arguments for further clinical characterization of SAHUE cases. Clinical characterization of SAHUE could reveal conditions relating HAdV and AAV2 viruses to the observed incidence of acute hepatitis. In addition, wastewater sampling could aid in surveillance for HAdV-F, AAV2, and other respiratory adenovirus species that might be involved in SAHUE outbreaks.

Appendix Additional information on adeno-associated virus 2 and human adenovirus F41 in wastewater during outbreak of severe acute hepatitis in children, Ireland.
