# Supplementary material for: Adeno-Associated Virus 2 and Human Adenovirus F41 in Wastewater during Outbreak of Severe Acute Hepatitis in Children, Ireland
Source: Emerg Infect Dis. 2023 Apr;29(4):751–60. doi: 10.3201/eid2904.221878 (PMC10045691; doi:10.3201/eid2904.221878)
Supplement: Appendix — Additional information on adeno-associated virus 2 and human adenovirus F41 in wastewater during outbreak of severe acute hepatitis in children, Ireland. [file 22-1878-Techapp-s1.pdf]

Article DOI: https://doi.org/10.3201/eid2904.221878

Adeno-Associated Virus 2 and Human Adenovirus F41 in Wastewater during Outbreak of Severe Acute Hepatitis in Children, Ireland

Appendix

Additional Materials and Methods

Calculation of Limit of Detection and Limit of Quantification for qPCR

The amplification efficiency of each assay was calculated with the slope of the standard curve using the equation $E = 10^{(-1/\text{slope})}$ (*1*). All reaction efficiencies were between 90 and 110%. The limit of quantification (LOQ) was specified as the lowest concentration of DNA quantified within 0.5 standard deviations of the $\log_{10}$ concentration, and the limit of detection (LOD) was specified as the lowest concentration of DNA detected in 95% or more of replicates (*2*,*3*).

Nanopore Sequencing of HAdV Hexon Subgenomic Fragments from Wastewater

200 fmol of amplicons were end-repaired by the NEBNext Ultra II End Repair/dA-Tailing Module (New England Biolabs, https://www.neb.com) with 1.75 µl of buffer and 0.75 µl enzyme made up to 15 µl with nuclease-free water and incubated at 20°C for 15 min and 65°C for 15 min. Native barcodes were ligated to the amplicons employing 5 µl of NEB Blunt/TA Ligase Master Mix with 0.9 µl of end-prepped DNA, 1.25 µl of individual barcode for each sample made up to 10 µl and incubated at 20°C for 20 mins and at 65°C for 10 mins. The barcoded library was pooled and mixed with 0.4 volumes of AMPure XP magnetic beads (Beckman Coulter, https://www.beckmancoulter.com) and incubated on a HulaMixer for 30 minutes. The pellet was spun down and separated on a magnetic rack for 5 mins, then washed twice in Short Fragment Buffer (SFB; Oxford Nanopore Technologies, https://nanoporetech.com), then washed with 100 µL 80% [v/v] ethanol, and finally eluted in 30 µl of EB (Oxford Nanopore Technologies) and quantified on a Qubit fluorometer. Adaptor

ligation was performed with the NEBNext Quick Ligation Module (New England Biolabs) by incubating the entire barcoded library with 5 µl of Adaptor Mix II (AMII), 10 µl of NEBNext Quick Ligation Reaction Buffer and 5 µl of Quick T4 DNA Ligase (New England Biolabs) in a 50 µl reaction volume and incubated at room temperature for 10 mins. Twenty µl of AMPure XP beads were added and incubated on a HulaMixer for 30 minutes, then washed twice in 125 µl of SFB and finally eluted in 15 µL of EB and quantified. Fifteen ng of the prepared library was loaded onto a FLO-MIN106D flow cell (Oxford Nanopore Technologies). Data acquisition was executed for 36 hours and subsequently called bases and demultiplexed with Guppy Basecalling Software version 6.3.7 (Oxford Nanopore Technologies), and adapters were trimmed with porechop version 0.3.2pre (https://github.com/rrwick/Porechop).

References

1. Rutledge RG, Côté C. Mathematics of quantitative kinetic PCR and the application of standard curves. Nucleic Acids Res. 2003;31:e93. PubMed https://doi.org/10.1093/nar/gng093

2. Blanchard P, Regnault J, Schurr F, Dubois E, Ribière M. Intra-laboratory validation of chronic bee paralysis virus quantitation using an accredited standardised real-time quantitative RT-PCR method. J Virol Methods. 2012;180:26–31. PubMed https://doi.org/10.1016/j.jviromet.2011.12.005

3. Rutledge RG, Stewart D. Critical evaluation of methods used to determine amplification efficiency refutes the exponential character of real-time PCR. BMC Mol Biol. 2008;9:96. PubMed https://doi.org/10.1186/1471-2199-9-96

4. Tiemessen CT, Nel MJ. Detection and typing of subgroup F adenoviruses using the polymerase chain reaction. J Virol Methods. 1996;59:73–82. PubMed https://doi.org/10.1016/0166-0934(96)02015-0

5. Centers for Disease Control and Prevention. 2019-novel coronavirus (2019-nCoV) real-time rRT-PCR panel primers and probes [cited 2022 Sep 29]. https://www.cdc.gov/coronavirus/2019-ncov/downloads/rt-pcr-panel-primer-probes.pdf

6. García-Aljaro C, Ballesté E, Muniesa M, Jofre J. Determination of crAssphage in water samples and applicability for tracking human faecal pollution. Microb Biotechnol. 2017;10:1775–80. PubMed https://doi.org/10.1111/1751-7915.12841

Appendix Table 1. Oligonucleotide primers and probes and thermocycling parameters for qPCR and dPCR assays*

Target	Primer and probe sequences, 5′-3′	PCR type	Primer, probe concentration	LOD/LOQ slope, efficiency	Thermocycling conditions	Reference
HAdV-F		qPCR		7.6 GC/µL −3.3142 100.3%	95°C, 120 s, 40 cycles at 95°C, 15 s; 60°C, 30 s	(*4*)
Forward	CACTTAATGCTGACACGGGC		300 nmol			
Reverse	ACTGGATAGAGCTAGCGGGC		300 nmol			
Primer	FAM-TGCACCTCTTGG ACTA GT-IBFQ		100 nmol			
AAV2 VP1		qPCR			50°C, 120 s; 95°C, 20 s; 40 cycles at 95°C, 3 s; 60°C 30 s	RIVM†
Forward	TACCTCAAGTACAACCAC GC		400 nmol			
Reverse	CCTCTTTTTSGCCTG GAAGA		400 nmol			
Primer	FAM-ATACGTCTTTTGGGGGC AACCTCG – BHQ-1		100 nmol			
AAV2 VP1		dPCR			95°C, 120 s; 40 cycles at 95°C, 5 s; 60°C, 30s	RIVM†
Forward	TACCTCAAGTACAACCAC GC		600 nmol			
Reverse	CCTCTTTTTSGCCTG GAAGA		600 nmol			
Primer	FAM-ATACGTCTTTTGGGGGC AACCTCG – BHQ-1		200 nmol			
AAV2 NSP		qPCR			50°C, 120 s; 95°C, 20 s; 40 cycles at 95°C, 3 s; 60°C, 30 s	RIVM†
Forward	AAGGTCACCAAGCAGGAAGT		400 nmol			
Reverse	CGTTTGGGCTCACTTATATCTG		400 nmol			
Primer	FAM-ACCCCGCATTACGTTTGG TGGACC - IBFQ		100 nmol			
AAV2 NSP		dPCR			95°C, 120 s; 40 cycles at 95°C, 5 s; 60°C, 30 s	RIVM†
Forward	AAGGTCACCAAGCAGGAAGT		600 nmol			
Reverse	CGTTTGGGCTCACTTATATCTG		600 nmol			
Primer	FAM-ACCCCGCATTACGTTTGG TGGACC - IBFQ		200 nmol			
SARS-CoV-2 N1		qPCR		5 GC/µL; −3.2177 99%	RT: 50°C, 600 s; 95°C, 30 s; 45 cycles at 95°C, 5 s; 60°C, 30 s	(*5*)
Forward	GACCCCAAAATCAGCGAAAT		500 nmol			
Reverse	TCTGGTTACTGCCAGTTGAATCT G		500 nmol			
Primer	FAM- CTACGTCAAAAAGGGTGGA GC- BHQ-1		125 nmol			
crAss_2		qPCR		1.56 GC/µL; −3.3068 99.4%	40 cycles at 95°C, 15 s; 60°C, 60 s	(*6*)
Forward	AGGAGAAAGTGAACGTGGAAAC A		950 nmol			
Reverse	AACGAGCACCAACTTTAAGCTTT A		950 nmol			
Primer	FAM-AGGATTTGGAGAAGGAA-MGB		250 nmol			

*AAV2, adeno-associated virus type 2; BHQ-1, blackhole quencher-1; GC, genome copies; HAdVF, human adenovirus type F; IBFQ, Iowa black FQ quenchers; N1, nucleocapsid protein 1; NSP, nonstructural protein; RIVM, Rijksinstituut voor Volksgezondheid en Milieu Hygiene, the Netherlands
†AAV2-VP1 and AAV2-NSP oligonucleotide primer and probe sequences were kindly provided by Dr. Harry Vennema at RIVM, through the European Centre for Disease Control and Prevention EpiPulse portal.

Appendix Table 2. Human adenovirus hexon amino acid sequences used to identify HAdV types in the wastewater samples

No.	Accession No.	HAdV species	Type
1	BAG48789	A	12
2	NP_040924	A	12
3	BAG48808	A	31
4	CAO78638	A	31
5	AKL80488	B	3
6	BAG48780	B	3
7	AGT77341	B	7
8	BAG48784	B	7
9	AAN62515	B	11
10	AAP49209	B	11
11	BAG48788	B	11
12	QZA82869	B	11
13	ACO81799	B	14
14	AFK92966	B	14
15	BAG48791	B	14
16	BAG48793	B	16
17	BAG48798	B	21
18	BAB20014	B	34
19	BAG48811	B	34
20	AAP92351	B	35
21	AP_000585	B	35
22	BAB20015	B	35
23	BAG48812	B	35
24	BAG48827	B	50
25	AGT55553	B	55
26	AET87230	B	68
27	BAG48778	C	1
28	QDO16041	C	1
29	BAG48779	C	2
30	AP_000211	C	5
31	BAG48782	C	5
32	BAG48783	C	6
33	BAU36782	C	6
34	AHI45188	C	57
35	ANW61309	D	8
36	BAG48785	D	8
37	BAE66671	D	9
38	BAG48786	D	9
39	AFK92137	D	10
40	BAG48787	D	10
41	BAM66756	D	10
42	AFK92177	D	13
43	BAG48790	D	13
44	BAG48792	D	15
45	ADY18429	D	17
46	BAG48794	D	17
47	BAG48795	D	18
48	BAG48796	D	19
49	BAG66282	D	19
50	AFK92257	D	20
51	BAG48797	D	20
52	ACL13145	D	22
53	ACR78217	D	22
54	BAG48799	D	22
55	AFK92297	D	23
56	BAG48800	D	23
57	AFK92336	D	24
58	BAG48801	D	24
59	AFK92376	D	25
60	BAG48802	D	25
61	ABO61316	D	26
62	BAG48803	D	26
63	AFK92416	D	27
64	BAG48804	D	27
65	BAG48805	D	28
66	AFK92456	D	29
67	BAG48806	D	29
68	AEY79573	D	30

No.	Accession No.	HAdV species	Type
69	BAG48807	D	30
70	AFK92496	D	32
71	BAG48809	D	32
72	AFK92576	D	33
73	BAG48810	D	33
74	ACY04472	D	36
75	BAG48813	D	36
76	ABK59070	D	37
77	BAG48814	D	37
78	BAH19144	D	37
79	ABO47729	D	38
80	AFK92616	D	38
81	BAG48815	D	38
82	AFK92656	D	39
83	BAG48816	D	39
84	AFK92696	D	42
85	BAG48819	D	42
86	AFK92736	D	43
87	BAG48820	D	43
88	AFK92776	D	44
89	BAG48821	D	44
90	AFK92816	D	45
91	BAG48822	D	45
92	BAG48823	D	46
93	AFK92536	D	47
94	BAG48824	D	47
95	BAG48825	D	48
96	BAG48826	D	49
97	AFK92856	D	51
98	BAG48828	D	51
99	BBW89580	D	53
100	YP_003038612	D	54
101	AGM61353	D	56
102	ADW95419	D	58
103	AEI91289	D	59
104	AEL78878	D	60
105	AFA46691	D	64
106	AFK92217	D	69
107	AGT76762	D	71
108	BAO53845	D	81
109	AZI15535	E	4
110	BAG48781	E	4
111	BBH49391	E	4
112	AAC13967	F	40
113	BAG48817	F	40
114	CAA36077	F	40
115	ACH90432	F	41
116	BAG48818	F	41

*HAdV, human adenovirus.

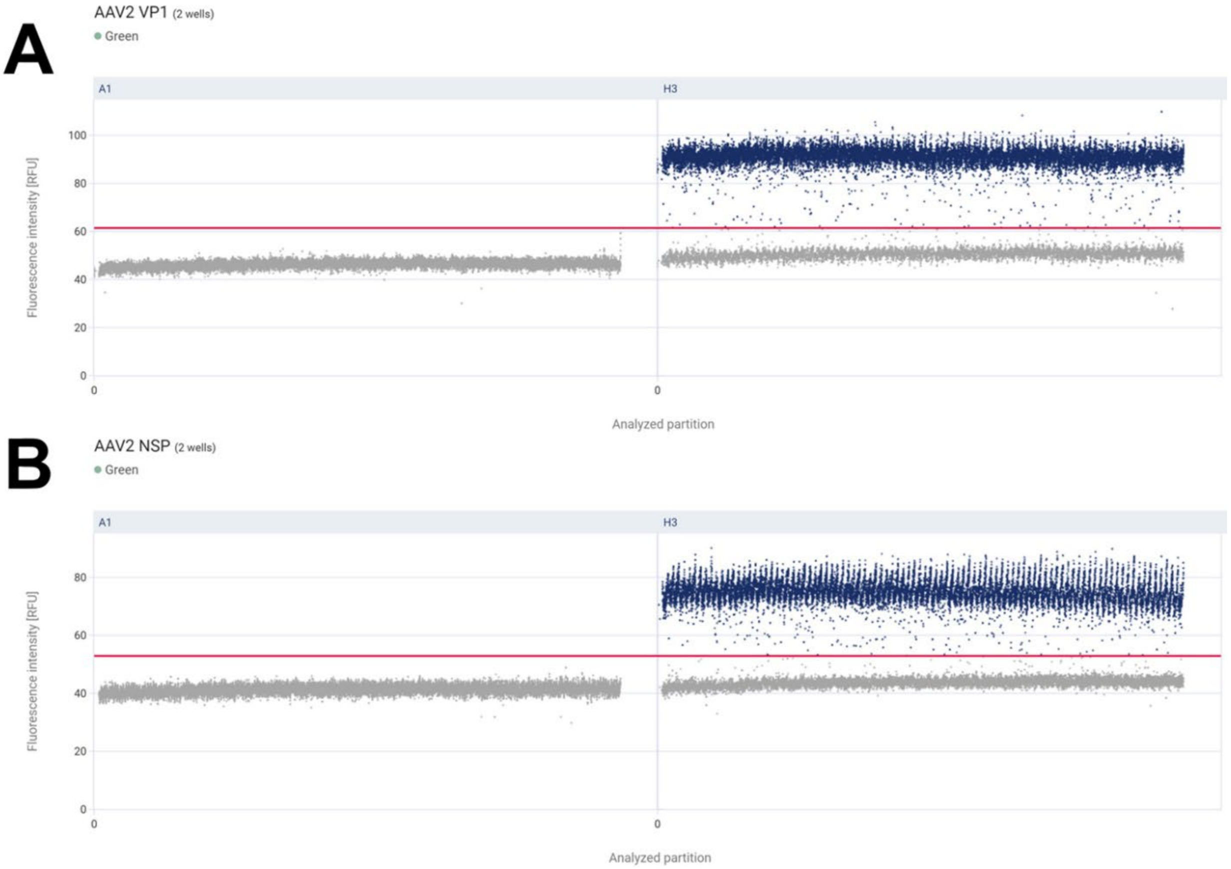

Appendix Figure 1. Adenovirus-associated virus 2 genes detected in wastewater, Ireland. A) AAV2 VP1; B) AAV2 NSP. Genes were detected by dPCR assays using nuclease free water as negative control (left panels; A1) and AAV2–positive plasma clinical sample as the positive control (right panels; H3). AAV2, adeno-associated virus type 2; N1, nucleocapsid protein 1; NSP, nonstructural protein.

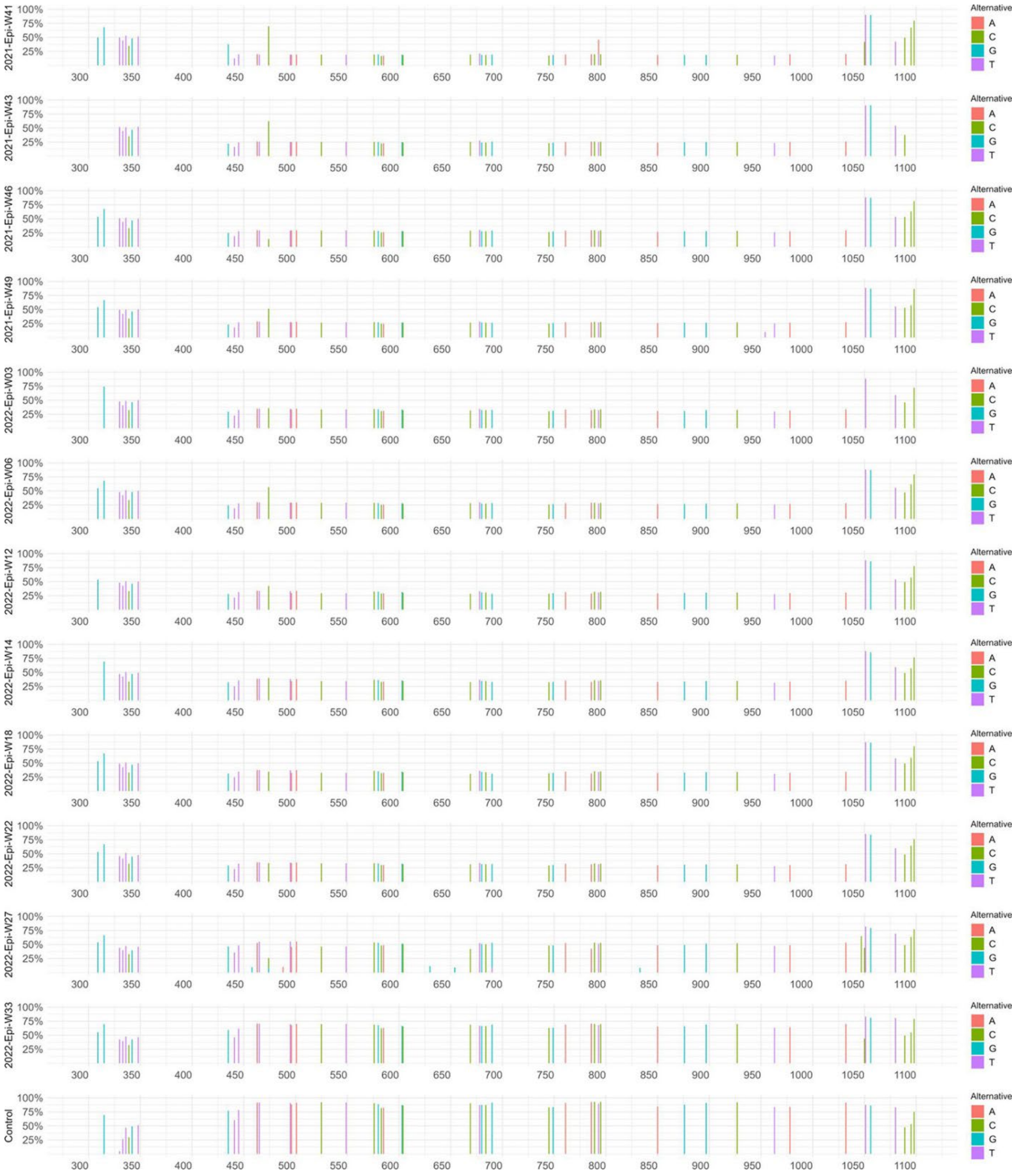

Appendix Figure 2. Human adenovirus type F41 (HAdV-F41) single nucleotide variant frequencies per wastewater sample, Ireland. The 13 panels correspond to the samples from each epidemiology week, as indicated on the left of each panel. The vertical axes indicate the frequency of the SNV in each sample and the horizontal axes are aligned across panels displaying the position relative to the hexon nucleotide position in the HAdV-F41 (GenBank accession no. ON442316). The bars representing the SNVs are color-coded according to the alternative nucleotide found in the reads.
